# Submarine outfall relocation and the reduction of microbiological risk in bathing waters

**DOI:** 10.1007/s11356-026-37759-z

**Published:** 2026-04-24

**Authors:** Renato Castiglia Feitosa, Paulo Cesar Colonna Rosman

**Affiliations:** 1 Department of Sanitation and Environmental Health of the, National School of Public Health, Oswaldo Cruz Foundation, Rio de Janeiro, Brazil; 2 Department of Coastal and Oceanographic Engineering, Federal University of Rio de Janeiro, Rio de Janeiro, RJ Brazil

**Keywords:** Submarine outfalls, Bacterial decay, Water quality, Coupling models, *E. coli* plumes, Coastal pollution

## Abstract

Urbanisation of coastal zones increases sewage loads and microbiological risks in bathing waters. This study evaluates whether relocating a planned submarine outfall can reduce these risks while preserving suitable bathing conditions along the Itaipuaçu coast, Rio de Janeiro, Brazil. A coupled framework integrates three-dimensional hydrodynamics, near-field plume simulations, and a Lagrangian far-field transport model with an *E. coli* decay formulation driven by solar radiation, temperature, and salinity. Hydrodynamic fields forced by tides, large-scale circulation, and local and remote winds were validated against in situ current measurements during winter conditions. Deterministic simulations and a probabilistic analysis quantified *E. coli* concentrations and the percentage of time that bathing-water thresholds (800 MPN/100 ml) are exceeded in the vicinity of the outfall diffuser and along the shoreline. Results show that an alternative outfall location eliminates plume contact with Maricas Islands, maintains compliant shoreline bathing waters, and reduces outfall length, providing a transferable framework for optimising regional submarine outfall design.

## Introduction

Coastal zones have undergone significant urbanisation over the last few decades, leading to changes in local ecosystems and to water quality degradation unless sanitation measures are implemented (Rangel-Buitrag et al. 2024). Conventional wastewater treatment plants can reduce pathogen concentrations in beach zones through a post-disinfection process, such as chlorination or ultraviolet radiation. However, these technologies often require large infrastructure, high energy demand, and operational costs (Gonçalves & De Souza 1997). In addition, disinfection processes may lead to the formation of potentially harmful disinfection by-products (DBPs) in treated effluents (Liao et al. 2024; Zhong et al. 2024); Shekhawat et al. 2026). Thus, submarine outfalls can provide an alternative due to the low risk of contaminating bathing zones through turbulent sewage dispersion and microbiological degradation (Besley and Birch 2019; Masria et al. 2024). The survival of faecal indicator bacteria, such as *Escherichia coli*, in seawater is controlled by interactions among physical, chemical, and biological processes. Recent work on marine and coastal systems shows that temperature, solar radiation, and salinity govern *E. coli* decay dynamics and should be explicitly considered in water quality and predictive models (Jozić & Šolić, 2017; Wong et al. 2022; Desta et al. 2024). Among these, solar radiation, temperature, and salinity are the primary environmental parameters governing decay rates. Although additional mechanisms, such as predation and sedimentation, may also influence *E. coli* decay rates, these processes are challenging to quantify and to incorporate into the modelling process (Chamberlin 1978; Fujioka et al. 1981). However, it is essential to highlight that the microbiological degradation in the marine environment occurs mainly due to the penetration of solar radiation along the water column (Chan et al. 2015; Guillaud et al. 1997; Sarikaya & Saatçi, 1995; Šolić & Krstulović, 1992; Suzuki et al. 2021). Studies in tropical and temperate coastal waters show that higher salinity and UV levels generally accelerate *E. coli* decay in seawater, while site-specific conditions modulate these effects (Wong et al. 2022; Desta et al. 2024). In coastal environments, the vertical position of the sewage plume within the water column is critical, as it governs the extent of light exposure to and, consequently, the spatiotemporal pattern of *E. coli* decay.

Recreational water quality assessments rely heavily on faecal indicator bacteria to infer health risks associated with primary contact. While some guidelines consider only Enterococcus as an indicator in marine waters (Health Canada 2023; United States Environmental Protection Agency 2012), the Brazilian and the European guidelines (CONAMA, 2000; European Union 2006) also consider *E. coli* due to its high density in domestic sewage and its reliability in detecting recent faecal contamination events in coastal areas where sewage is the primary source of pollution. In addition, studies along the Brazilian coast found no differences in bathing water quality standards when using either *E. coli* or Enterococcus as faecal indicators (Castiglia Feitosa and  Rosman 2024; Pedrosa de Macena et al. 2023; Schaffel et al. 1990).

It is worth highlighting that deterministic models provide instant plume concentration, but do not provide the frequency and duration of non-compliance events. On the other hand, probabilistic modelling enables a risk-based assessment by quantifying the percentage of time water quality standards are exceeded, a critical metric for long-term environmental management and public health.

Numerical modelling has become a valuable tool for planning, designing, and optimising submarine outfalls, integrating hydrodynamic circulation and near-field and far-field transport modelling (Feitosa et al. 2013; Morelissen et al. 2013; Castro Faccetti 2020; Porto Pereira et al. 2024). A coupling model’s methodology enables the exploration of realistic scenarios under varying hydrodynamic and metocean conditions that govern *E. coli* die-off in marine waters, including the linkage between near-field, far-field, and water quality modules (Castro Faccetti 2020) Despite the relevance of submarine outfalls as a sanitation solution for coastal areas, relatively few studies combine near-field/far-field coupling, which explicitly accounts for solar-radiation effects on *E. coli* decay, with a probabilistic assessment of bathing-water risk along beach zones.

In this context, the present study aims to evaluate alternative locations for a submarine outfall at Itaipuaçu, on the coast of Rio de Janeiro State, using a coupled scheme that integrates hydrodynamics, near-field and far-field models, linked to an *E. coli* decay model driven by solar radiation, temperature, and salinity (Rosman 2024). Specifically, the study aims to (i) validate the hydrodynamic field in the study area, by comparing measured and computed current data; (ii) evaluate the *E. coli* concentration plumes under simultaneous metoceanographical conditions; and (iii) compare two different locational options for sewage discharge, evaluating the risk of exceeding microbiological bathing water limits at nearby beaches and islands.

## Methodology

### Study area and model domain

**Fig. 1:**
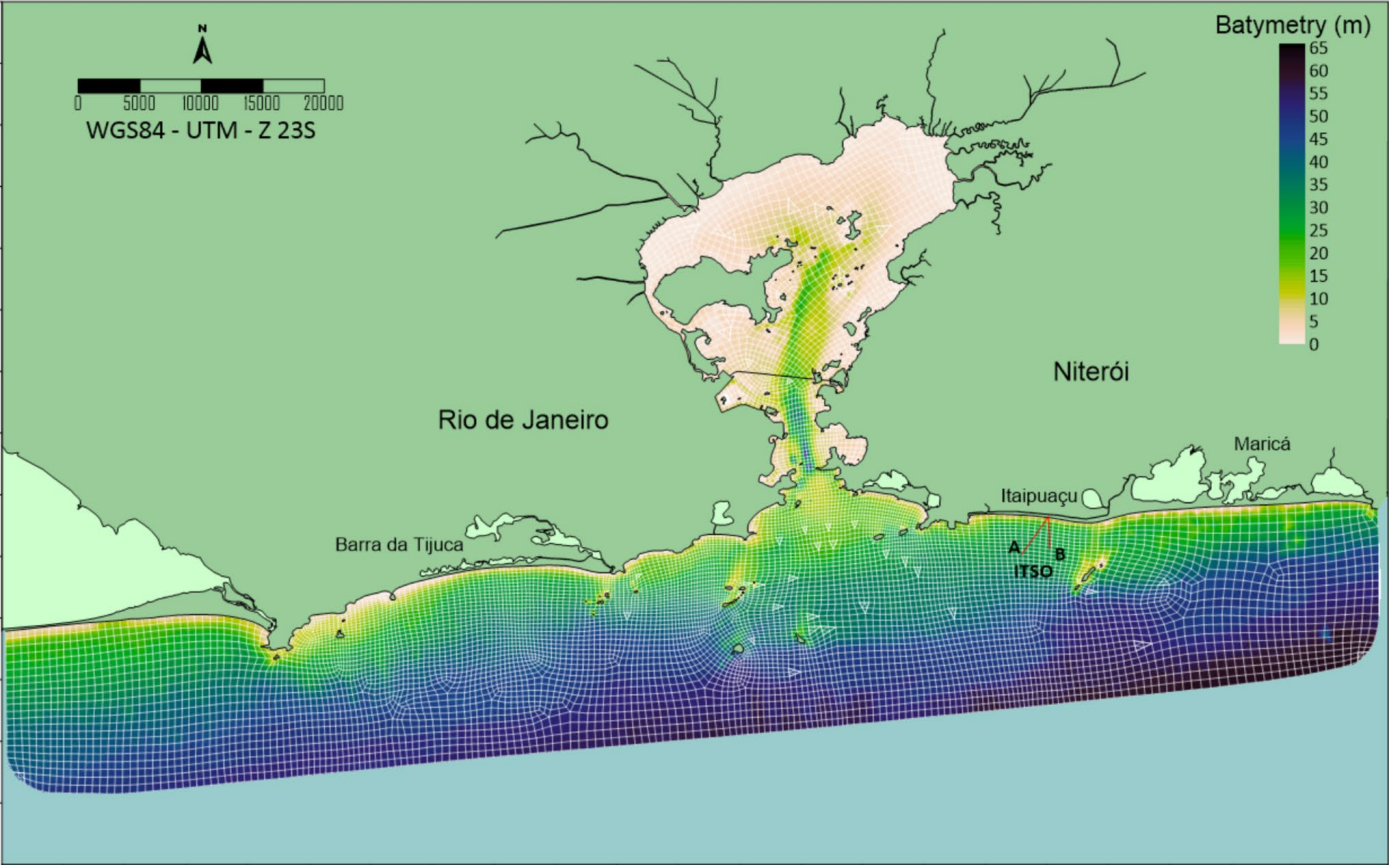
Bathymetry of the area of study and the position of Itaipuaçu submarine outfall

Figure 1 shows the depth contours and the alternative locations for the outfall discharge point of the future Itaipuaçu submarine outfall (ITSO) located in Niterói city (Rio de Janeiro state, Brazil): the original location (A) with a 3715 m pipeline length, and an alternative (B) with a 2540 m pipeline length. This figure also includes the bathymetry and the discretising mesh used in the modelling. The ITSO is designed to discharge up to 600 l/s of domestic sewage through a 710-mm-diameter high-density polyethene (HDPE) pipeline at a depth of approximately 30 m. The diffuser is 34 m long and has 30 nozzles (75 mm diameter) spaced 2.4 m apart. Before submarine launch, the effluent is primarily treated, resulting in an *E. coli* concentration of 3.5 × 10^7^ MPN/100mL, with an efficiency of 30% at this treatment level (Raboni et al. 2016).

The discretisation of the modelled domain is performed using a geometric mesh with 10,993 nodes, comprising 2637 quadrangular and triangular elements across 11 vertical levels, resulting in 29,007 elements and 120,923 calculation points. The horizontal resolution of the mesh was optimised for computational efficiency and accuracy, ranging from 300 to 400 m in the vicinity of the outfall diffuser and along the shoreline, and from 600 to 700 m in the inner part of the bay and near the open boundaries. The discharge depth of 30 m was selected to ensure high initial dilution due to buoyancy effects, considering two different pipeline/diffuser conditions: axis (A) farther from the Maricas Islands and the coast; axis (B) closer to the Maricas Islands and the coast. This discharge depth is based on the fact that the initial dilution is primarily governed by the vertical distance available for buoyant plume rise, where the entrainment between the effluent plume and the seawater is directly proportional to the discharge depth. A depth of 30 m provides a significant vertical trajectory, ensuring that the plume undergoes extensive turbulent mixing and reaches a high dilution factor before it reaches its maximum rise height.

As shown in Fig. 1, bathymetry contours were derived from Brazilian Navy nautical data available on the Brazilian Navy website (Directorate of Hydrography and Navigation, 2022). The bottom sediment that composes the bed rugosity of the region ranges from fine to coarse sand, with silt pockets in exposed areas and sand and fine muddy sediments in the inner part of the bay (Kjerfve et al. 2021).

### Modelling process

**Fig. 2:**
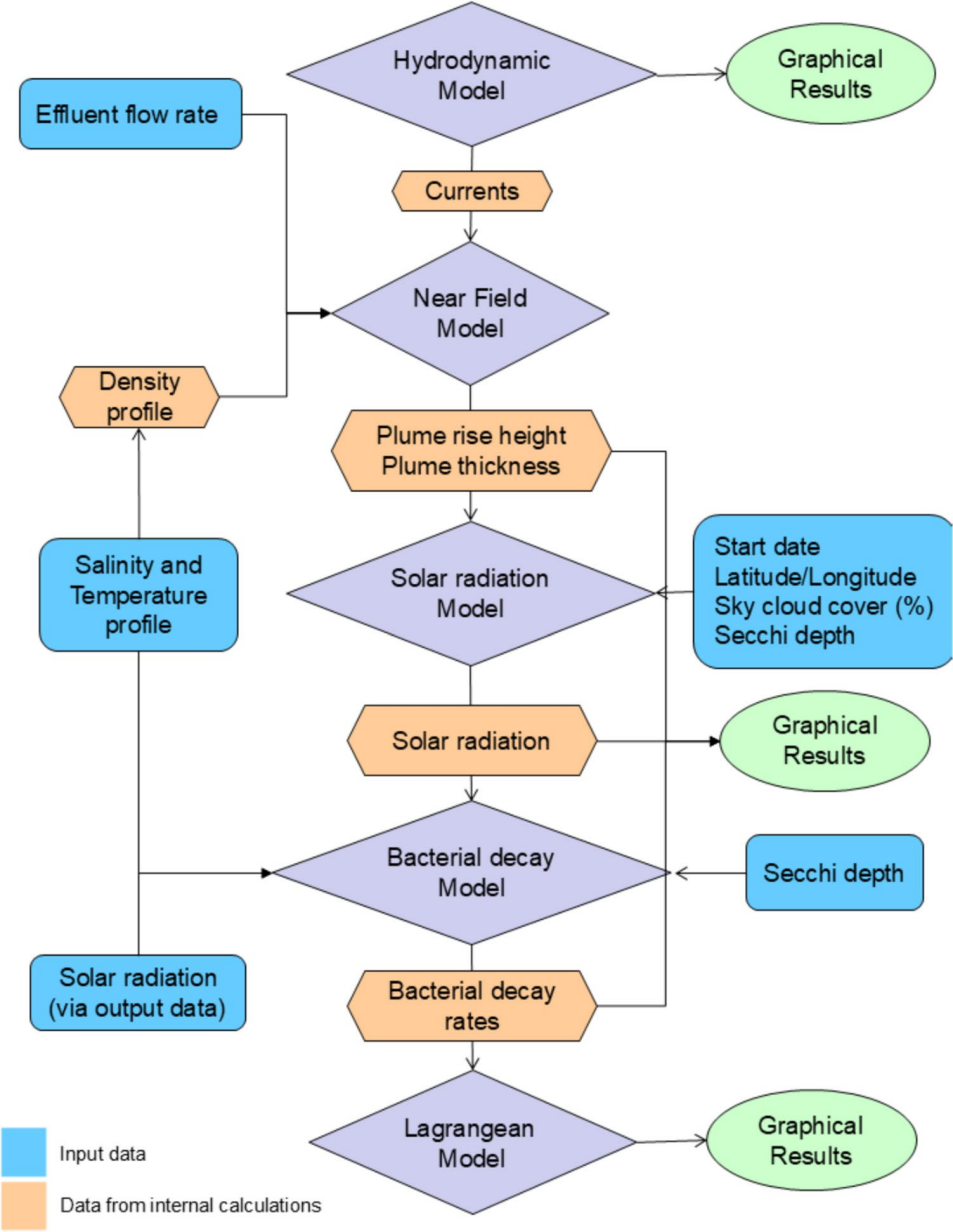
Scheme of coupling methodology between near-field and far-field models

Figure 2 presents the coupling scheme implemented in the SisBaHiA model, illustrating the stages among the hydrodynamic, near-field, far-field, and bacterial-decay models. Additional details about this coupling scheme are presented in Feitosa et al. (2013). SisBaHiA has been routinely used in water quality assessment in water bodies over the last few years (Demoner et al. 2023; Lopes de Barros et al. 2024; Trog-Ferreira et al. 2025). The coupling procedure aims to incorporate the dynamic characteristics of the marine environment, first by accounting for hydrodynamic behaviour and, second, by accounting for mixing between sewage and seawater and for *E. coli* decay.

#### Hydrodynamic model

The three-dimensional hydrodynamic model was implemented using SisBaHiA (Base System of Environmental Hydrodynamics), which solves the shallow-water equations (Navier–Stokes with hydrostatic pressure assumption) using the finite element method with a second-order time scheme and quadratic spatial discretisation (Rosman 2024). The current profiles depend on frictional forces, represented by wind stress at the free surface and bottom roughness. The turbulent stress is parametrised using filtering techniques (e.g., large-eddy simulation). The model simulates 31 days of winter conditions in the Southern Hemisphere (1–31 July 2022), representing a conservative scenario for bacterial decay due to reduced solar radiation. Regarding the forcing data and boundary conditions, tidal constituents were obtained from the FES2014 global model (Finite Element Solution; https://datastore.cls.fr/catalogues/fes2014-tidemodel/). Mean sea level and residual currents were derived from HYCOM (Hybrid Coordinate Ocean Model; www.hycom.org) and wind forcing from ERA5 reanalysis (ECMWF; https://cds.climate.copernicus.eu/). Meteorological tides play a dominant role relative to astronomical tides in the ITSO region, resulting in cyclical east–west current reversals with periods of 2–8 days. Open boundary conditions are specified for the sea-surface elevation and the depth-averaged residual currents. The hydrodynamic model was validated by comparing modelled and observed current velocities at a nearby location (the vicinity of the Barra da Tijuca submarine outfall) over 30 days in April 2008. This validation is appropriate since the Barra da Tijuca site shares the same inner-shelf dynamic regime and bathymetric orientation as Itaipuaçu, being subjected to identical wind-forcing patterns (NE/SW) and large-scale current influences. The 2008 dataset provides a robust, high-frequency ADCP record that accurately represents the region's characteristic response to synoptic meteorological events, ensuring the model’s reliability for the study area. Depth-averaged modelled currents showed good correlation with field data, confirming that the model adequately captures the local wind-driven current pattern.

#### Near-field modelling

**Fig. 3:**
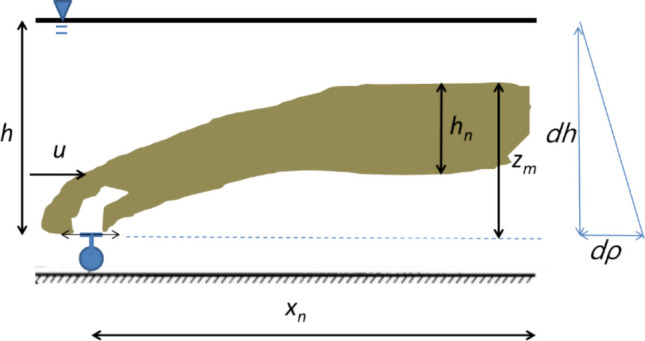
Plume characteristics in the near-field

The near-field model simulates the initial mixing of the effluent jet with ambient seawater as it rises along the water column and moves away from the discharge point. Using the NRFIELD methodology (Daviero & Roberts 2006; Roberts et al. 1989a, 1989b, 1989c; Tian et al. 2006, 2004a, 2004b), plume rise height (*z*_*m*_), thickness (*h*_*n*_), and dilution are calculated as functions of jet momentum, buoyancy, and ambient current velocity and density profile. As shown in Fig. 3, these quantities determine the plume’s vertical position in the water column, which, in turn, regulates the mean light intensity experienced by the plume and, hence, the *E. coli* decay rate in the far-field. Plume characteristics are sensitive to the mixing of the effluent along the water column, which is governed by the density profile (dρ/dz) between the surface and the discharge depth. Density profiles were computed internally using the Eckart equation, with temperature and salinity provided by the HYCOM database. Seasonal and episodic density stratification in the region (Carvalho et al. 2002; Araújo et al. 2023) ranges from homogeneous (southwest winds) to highly stratified (northeast winds with upwelling), both of which were represented in the 31-day winter simulation.

#### Far-field and bacterial decay modelling

The far field comprises a neutral mixing zone where microbiological decay occurs while sea currents passively advect the sewage plume. The far-field modelling comprises a Lagrangian methodology where the contaminant is represented by particles, whose location (*P*) varies over time according to the current pattern *V* (*u,v,w*) generated by the hydrodynamic model, where H.O.T. (higher order terms) are neglected (Rosman 2024).

1 $${P}^{n+1}={P}^{n}+\Delta t V+\frac{\Delta {t}^{2}}{2!}\frac{dV}{dt}+H.O.T.$$

The initial contaminant concentration (*C*_*0*_) follows first-order decay kinetics with a coliform decay rate (*k*).

2 $${C}_{t}={C}_{0}{e}^{-kt}$$

Mancini’s formulation (Mancini 1978) was adapted to quantify the *E. coli* die-off along the water column (Feitosa et al. 2013).

3 $$k=\left[0.8+0.006 \times \left(100\frac{S}{36}\right)\times {1.07}^{\left(T-20\right)}+\frac{{I}_{0}}{{h}_{n}}{e}^{-{K}_{e}d}\left[1-{e}^{-{K}_{p}{h}_{n}}\right]\right]$$

where *S* is salinity (ppt), *T* is temperature (°C), *I*_*0*_ is incoming solar radiation at the free surface (cal/cm^2^·h), *h*_*n*_ is plume thickness, *d* is the plume depth, and *K*_*e*_ and *K*_*p*_ are light extinction coefficients for ambient water and within the plume, respectively. As shown in Eq. 4, these coefficients are related to Secchi depths (*S*_*de*_ and *S*_*dp*_) in metres*.*

4 $${K}_{e}=\frac{1.8}{{S}_{de}} ; {K}_{p}=\frac{1.8}{{S}_{dp}}$$

Due to the absence of field data in the surroundings of the Itaipuaçu submarine outfall, an average Secchi depth value for July (7.6 m) was considered based on data from a 1-year oceanographic campaign performed in Barra da Tijuca off the coast region (CEDAE 1988). This value represents a conservative average condition for winter-based conditions, given that a lower Secchi depth would reduce bacterial decay rates, potentially extending the plume’s influence. The light extinction coefficient, considering this Secchi Depth assumption, was about 0.24 m^−1^.

Regarding the *E. coli* plume spreading in the far field, while turbulence parameters capture energy at the grid scale, dispersion coefficients represent mixing due to subgrid-scale eddies and vertical velocity shear. The horizontal dispersion coefficients (*K*_*x*_, *K*_*y*_ = 1.0 m^2^/s) were selected based on the characteristic length scales of the inner shelf and follow conservative values used in previous regional studies. Vertical dispersion (*K*_*z*_) was dynamically calculated by the model’s turbulence scheme, accounting for the buoyancy effects and vertical shear observed in the 3D hydrodynamic field. Additional details concerning these parameters are described in Rosman (2024).

Mancini’s formulation (Mancini 1978) is driven by abiotic factors that vary over time and was previously used to simulate *E. coli* plumes from a sewage outfall in the coastal waters of Rio de Janeiro (Feitosa & Rosman 2024).

Considering solar radiation as one of the main parameters in the *E. coli* modelling, it can be provided to the model as input data (ERA5 database) or calculated by the model using the methodology presented in Martin et al. (2018). The input data for this methodology comprises meteorological, geographical, and seasonal data that govern solar radiation levels at the free surface, such as cloud cover, latitude, and date.

## Results

The results presented comprise the different stages of the modelling process, including the hydrodynamic, near-field, far-field, and bacterial-decay models. The hydrodynamic results encompass a calibration process that compares measured and computed current values. The results are presented as graphs and maps, accompanied by vectors and colour scales that represent the magnitudes of the parameters studied in the modelling.

### Hydrodynamic model validation

**Fig. 4:**
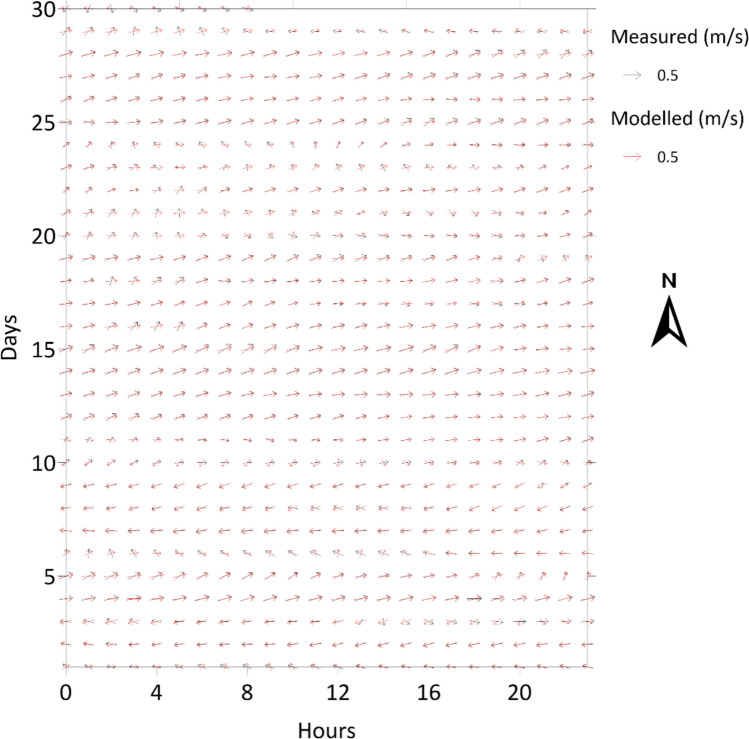
Comparison between modelled and field results. Currents measured around the vicinity of Barra da Tijuca submarine outfall in April 2008

The validation of the hydrodynamic model involves comparing modelled and field current data. A good agreement between the modelled and field data indicates that the model can adequately represent real conditions. In contrast, modelled results cannot be extrapolated to real conditions, and the model should be revised. Figure 4 presents a comparison between modelled and observed depth-averaged currents, near the Barra da Tijuca submarine outfall site over the 30-day April 2008 validation period, capturing both typical westward flows under prevailing easterly winds and eastward flows during south-westerly wind events, supporting the use of the simulated current fields in subsequent near-field and far-field analyses. The model validation showed a high level of agreement, with a combined vector root mean square error (RMSE) of 0.068 m/s, indicating close agreement with field conditions. Specifically, the east-west component (U) showed a strong correlation (*R* = 0.88; *R*^2^ = 0.77) and a low RMSE of 0.057 m/s. While the north-south component (V) presented a lower correlation (*R* = 0.63; *R*^2^ = 0.40), its absolute error remained very small (RMSE = 0.037 m/s), confirming that the model accurately reproduces both the magnitude and the predominant along-shore directional shifts of the currents.

### Hydrodynamic modelling

**Fig. 5:**
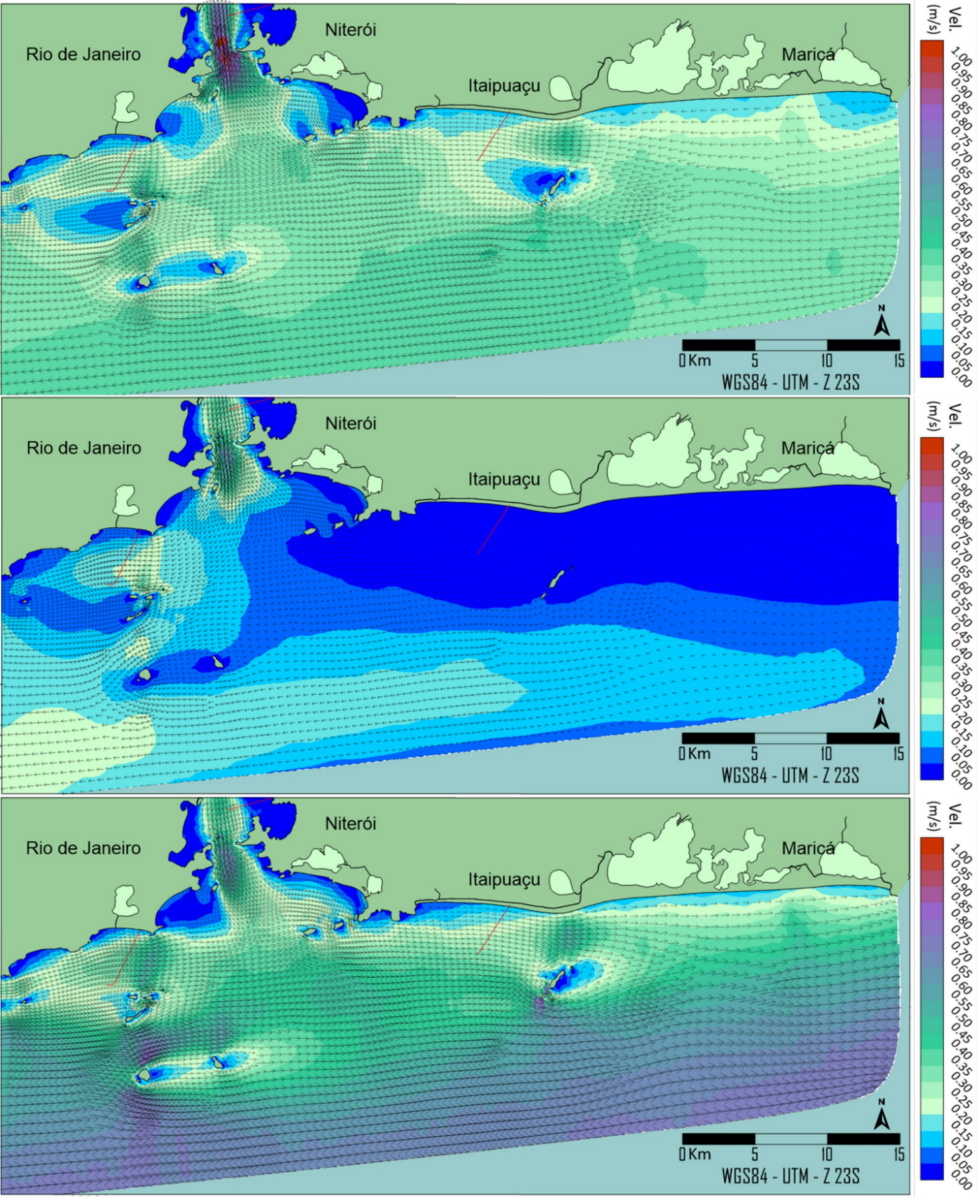
Hydrodynamic fields representing the current pattern in the studied area. **A** Currents flow west during good weather conditions. **B** Current field right before the inversion of the flow to the east. **C** Currents flow to the east during bad weather conditions

The hydrodynamic model encompasses winter conditions in the southern hemisphere, specifically the period from 1 to 31 July 2022. The maps in Fig. 5 depict different instants of the hydrodynamic field, with current intensity (m/s) represented by coloured isolines and current direction by vectors.

Current speeds in the ITSO region typically range from 0.1 to 0.5 m/s, with occasional peaks during strong south-westerly wind events. The flow alternated predominantly between westward and eastward directions in response to local and remote wind patterns, with negligible tidal modulation, and currents remained essentially unidirectional over the water column near the outfall.

### Near‑field modelling and bacterial decay rates

This part of the modelling encompasses the interdependence between the near-field model, which generates effluent plume characteristics such as rise height and thickness, and the far-field *E. coli* decay rates.

**Fig. 6:**
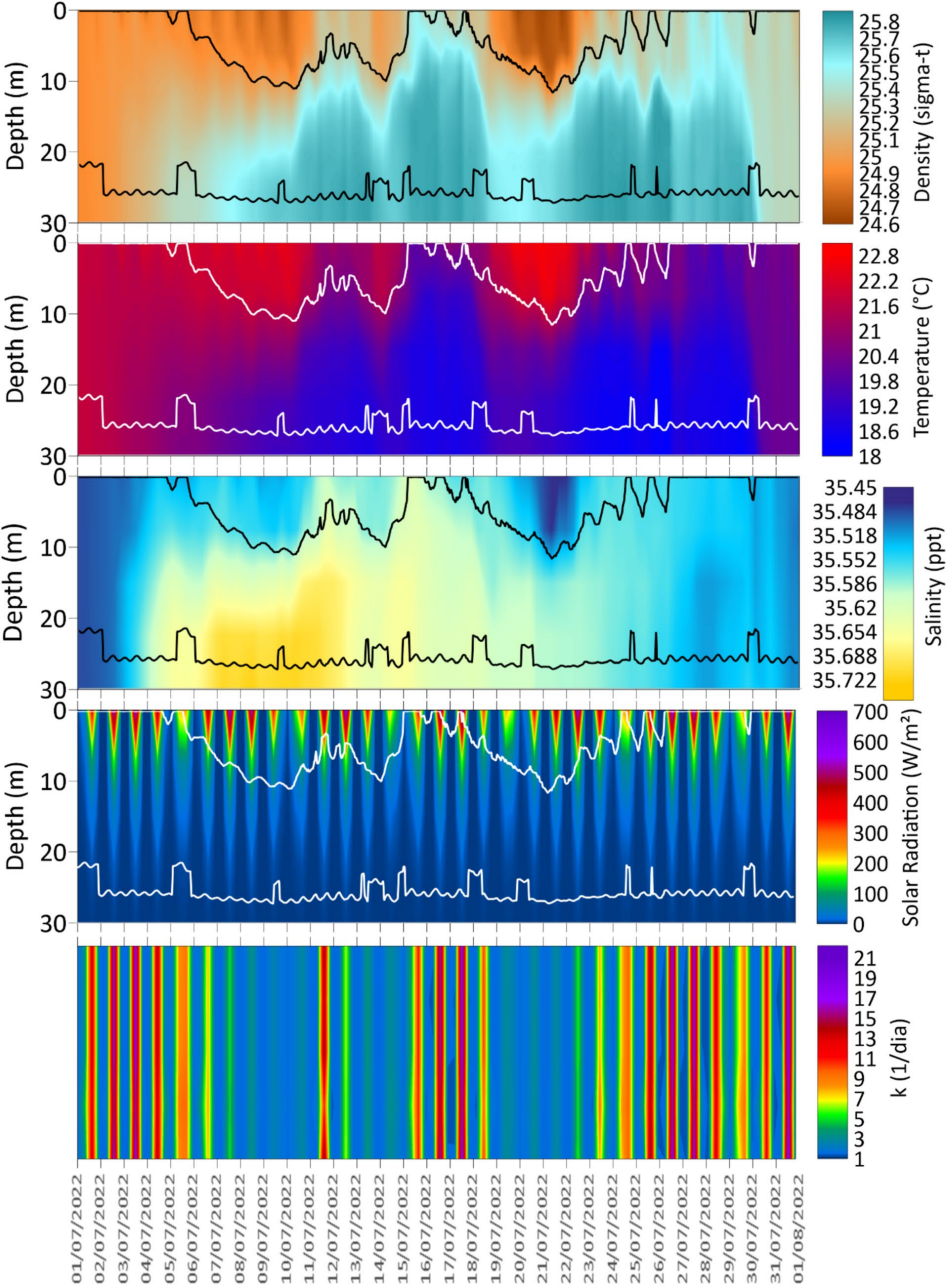
Time variation of density, temperature, salinity, and solar radiation along the water column and the averaged *E. coli* decay rate (k) along the plume thickness. The white and black lines indicate the upper and lower limits of the sewage plume in the water column over time

Figure 6 presents the *E. coli* decay (*k*) rates alongside the simultaneous variations in density, temperature, salinity, and solar radiation. Black and white lines represent the position of the sewage plume along the water column. The density profiles ranged from 24.6 to 25.8 sigma-t units, with temperature and salinity varying from 18.19 to 22.97 °C and from 35.47 to 35.72 ppt, respectively. Density differences along the water column regulate the plume’s depth. Higher density differences lead to submerged plume conditions, whereas lower density differences result in a surface sewage plume. As shown in Fig. 6, surface and submerged conditions alternate over the modelled period, regulating solar radiation along the plume thickness. The solar radiation provided by the ERA5 database comprises net radiation at the free surface, which ranges from 1.47 W/m^2^ in the early morning or late evening to 683 W/m^2^ at noon, accounting for cloud cover and other atmospheric factors that attenuate sunlight. Days and nights are clearly distinguished by colour scales, with deep blue indicating the absence of light. During the day, light extinction becomes evident in the water column, whose colours range from purple to light blue. Under such circumstances, the highest solar radiation at the free surface (683 W/m^2^) was substantially reduced to 0.56 W/m^2^ at 30 m depth.

The combination of averaged temperature, salinity, and solar radiation values yields *E. coli* decay rates along the sewage plume. A mean Secchi depth (7.6 m) was considered constant throughout the modelling period. During the daytime, the highest decay rate (21.07 day^−1^) occurred under the combination of higher radiation levels on the free surface with surface plume conditions, and the lowest decay rate (1.48 day^−1^) occurred due to simultaneous scenarios of lower solar radiation on the free surface and submerged plume. At night, in the absence of sunlight, *E. coli* decay rates depend on the temperature-salinity combination within the sewage plume in the water column, ranging from 1.35 to 1.67 day^−1^.

The decay rates directly affect the concentration of *E. coli* in sewage plumes as they move away from the effluent discharge point. In far-field modelling, lower concentrations are expected during episodes of higher decay rates, and vice versa.

### Far-field modelling

The following figures illustrate the *E. coli* concentration plumes under various oceanographic conditions, assuming two different sewage-release locations. As shown in Fig. 1, option A was the originally designed location for ITSO, with a total pipeline length of 3715 m. However, during the simulation, a trace of the effluent plume was evident near the ITSO diffuser in the Maricas Islands. Thus, an alternative pipeline axis of 2540 m, designated B, was proposed for the effluent discharge point to prevent the effluent from contacting the island, as mentioned earlier.

The results are presented for the two locational alternatives, using both deterministic and probabilistic approaches. In deterministic modelling, *E. coli* concentration plumes are depicted at different instants under varying metocean conditions. In contrast, the probabilistic approach presents the percentage of the modelling period during which *E. coli* concentrations are considered unsuitable for bathing (i.e., higher than 800 MPN/100 ml).

#### Deterministic modelling

**Fig. 7:**
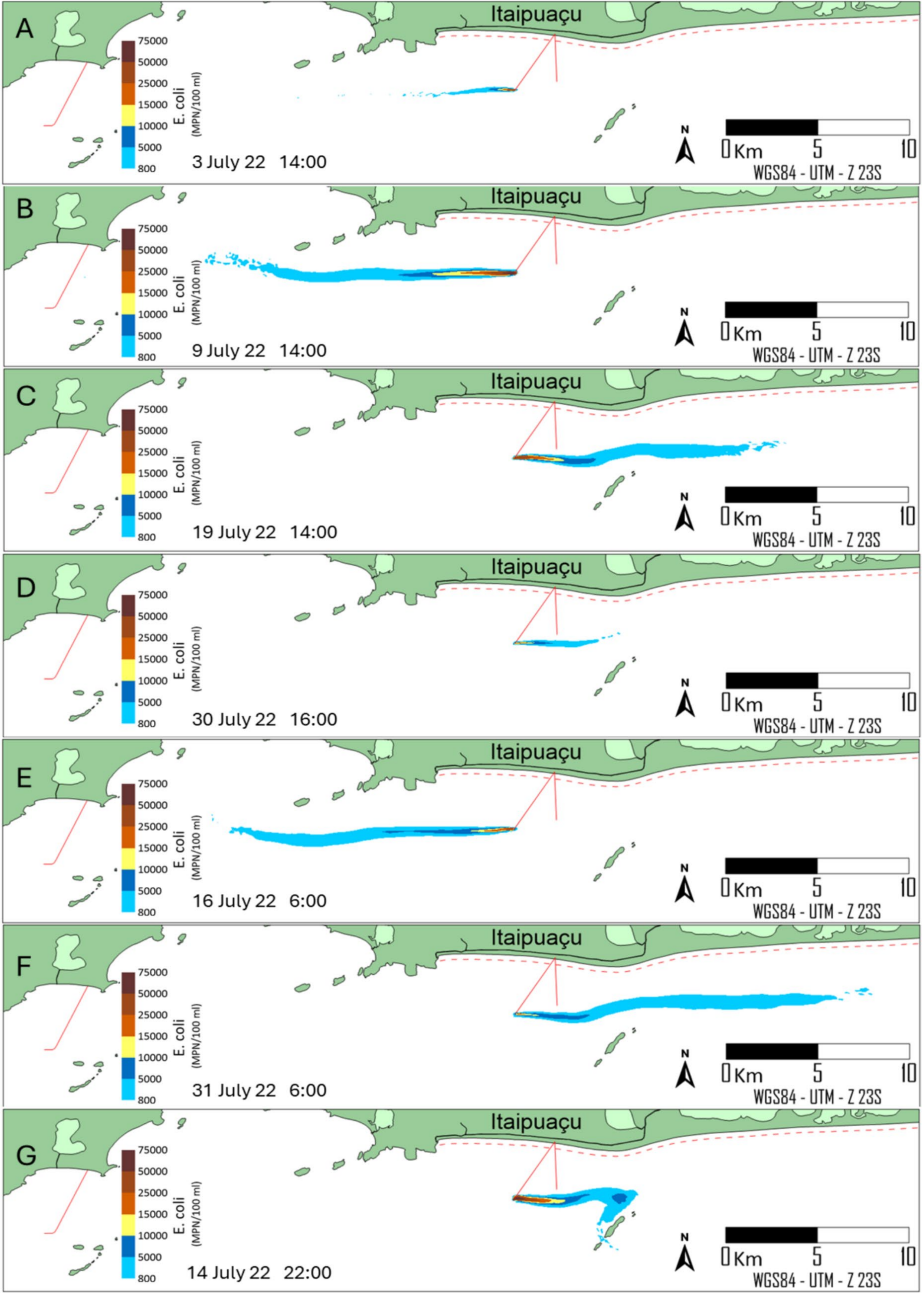
*E. coli* concentration levels at different moments under different metoceanographic conditions, for the original location of ITSO outfall. Concentrations above 800 MPN/100 mL indicate unsuitable bathing conditions. The red dotted line comprises the limit of bathing waters

**Fig. 8:**
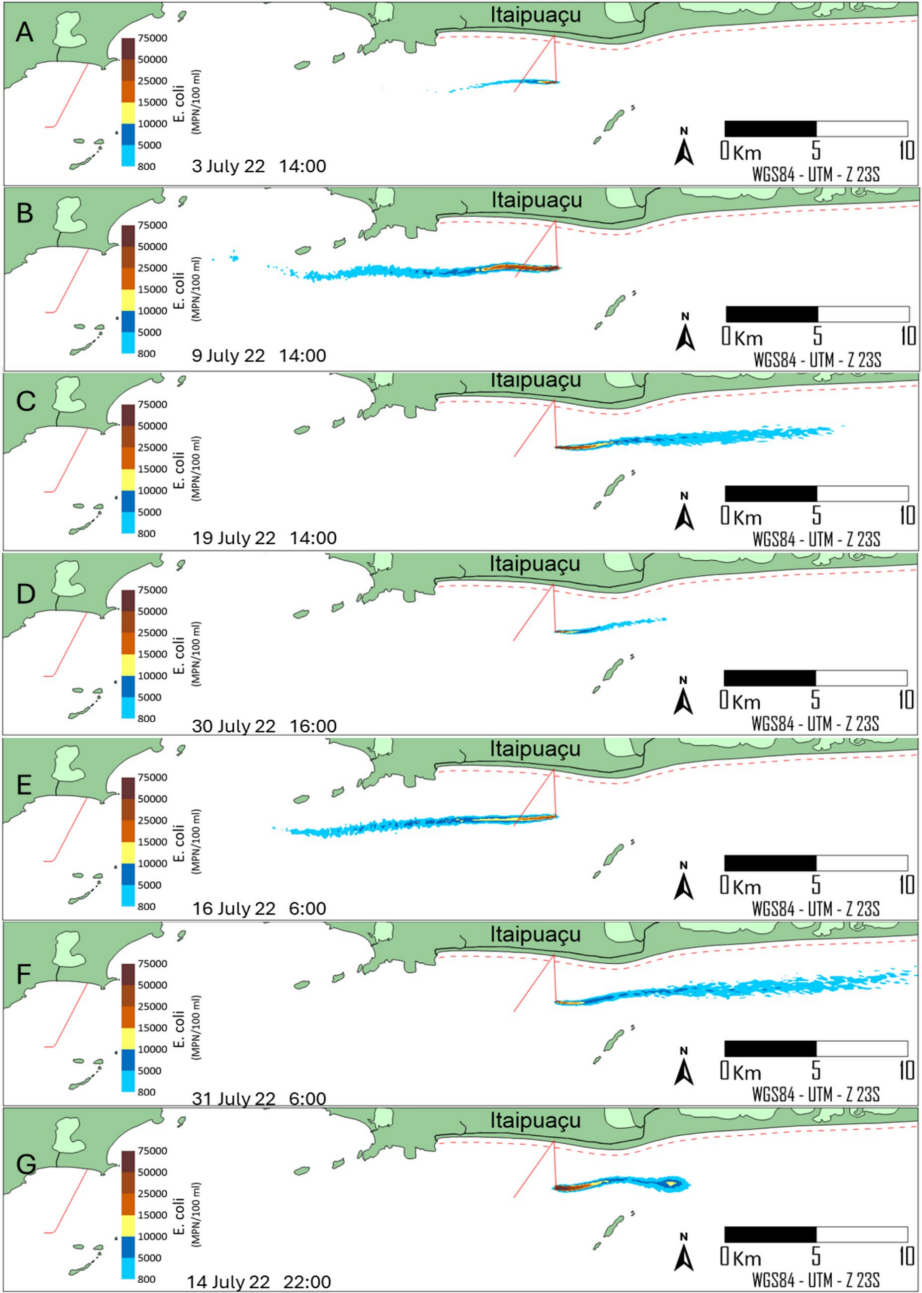
*E. coli* concentration levels at different moments under different metoceanographic conditions, for the alternative location of ITSO outfall. Concentrations above 800 MPN/100 mL indicate unsuitable bathing conditions. The red dotted line comprises the limit of bathing waters

It is essential to note that the modelling produces hourly results over 31 days, yielding 744 frames. Given the limited space, the most relevant moments were selected, with the remainder omitted. However, the probabilistic modelling presented in the following section spatially illustrates the overall aspects, showing the impact of the *E. coli* plume in the vicinity of the effluent discharge. Figures 7 and 8 present the *E. coli* concentrations for different locational options under the same time and metoceanographic conditions. These coloured scale concentrations illustrate unsuitable bathing conditions. Along with the currents, these concentrations are primarily influenced by daily variations in solar radiation across the plume thickness and by nighttime temperature and salinity.

In Fig. 7, under good weather conditions, currents at the original location flow from east to west. During adverse weather conditions, the currents reverse direction. On 3 July at 14:00 (frame A), during surface plume conditions, as highlighted in Fig. 6, higher solar radiation levels lead to shorter plume extensions, due to higher *E. coli* decay rates along plume thickness. Under the same radiation levels at the free surface and the same current pattern, submerged conditions on 9 July at 14:00 (frame B, 10 m depth) attenuate the sunlight incident on the plume, thereby increasing the *E. coli* concentration limits due to lower downstream decay rates. The same behaviour is observed under bad weather conditions, with longer *E. coli* concentration limits from the ITSO diffuser when comparing submerged (frame C, 7 m depth) to surface (frame D) plume conditions. Frames E (16 July at 6:00) and F (31 July at 6:00) represent, under different current patterns, surface *E. coli* plumes in the absence of solar radiation right before dawn (6 a.m.). High *E. coli* concentrations are observed during prolonged periods without sunlight, resulting in longer plumes under unsuitable bathing conditions. In addition, slight differences are observed between frames E and F, with higher concentrations on 16 July at 6:00 near the effluent discharge point, due to colder water conditions, which result in slower *E. coli* decay. Frame G (14 July 22:00, 5 m depth) indicates the touch of the *E. coli* plume in Maricas Islands.

Although the occurrence of the *E. coli* plume in Maricas Islands is rare, a second alternative location analysis with a shorter pipeline length is presented in Fig. 8, reproducing the same frames as the original location. As shown, the extent of *E. coli* plumes does not differ significantly from the original location because the two locations are proximal, resulting in similar hydrodynamic and decay-rate conditions. The alternative position eliminates the *E. coli* plume contact with Maricas Islands, providing a significant environmental benefit. It shortens the original pipeline length by 1175 m, yielding financial savings without compromising the water quality of the beach zone represented by the red dotted line. Even though the persistence of *E. coli* in marine sediments in recreational waters presents challenges for ecological and public health (Erb et al. 2024), the discharge of sewage sufficiently far from bathing areas does not pose a health risk.

#### Probabilistic modelling

**Fig. 9:**
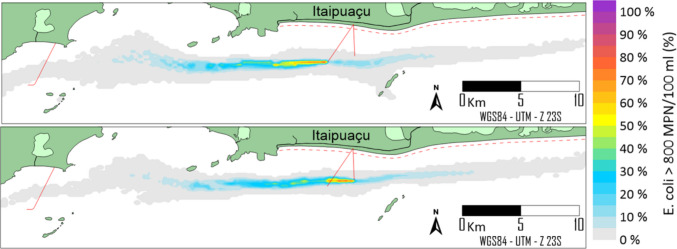
Probabilistic maps of *E. coli* plume behaviour over a 31-day winter cycle. The colour scale indicates the frequency (%) of time that concentrations exceeded the 800 MPN/100 mL threshold. **A** Original design location. **B** Optimised alternative location, showing the complete protection of the Marica’s Islands

Probabilistic modelling encompasses all the results of deterministic modelling within a single framework, providing a risk-based interpretation of the plume’s impact on coastal water quality, considering the frequency of unsuitable bathing conditions (*E. coli* > 800 MPN/100 ml). As shown in Fig. 9, the probability limits for the two alternatives did not differ significantly. The percentages are distributed parallel to the coast, not reaching the coastal zones. However, probabilities above 50% have shorter extensions in the alternative case because the effluent discharge depth is lower (28 m) than in the original (29 m). In the shallower case, the effluent is exposed to higher levels of solar radiation, which enhances the decay rates of *E. coli* and slightly reduces their concentration in marine waters.

It is also noted that the percentage of unsuitable bathing conditions aligns with the current direction, indicating a higher concentration of *E. coli* on the west side of the ITSO diffuser. Along this direction, percentages higher than 30% occur approximately 10 km away from the launching point. To the east of the launching point, percentages exceed 10% only up to 5 km, due to the lower incidence of currents in this direction, even during wintertime, when currents are more frequently from west to east.

The alternative location (alternative B) eliminated the risk of unsuitable bathing conditions at Maricas Islands during the entire simulation period. In contrast, the alternative A led to sporadic non-compliance events. This difference demonstrates that relocating the outfall not only reduces the pipeline length but also ensures a significantly higher safety margin for protected recreational zones.

## Discussion

Numerical modelling has become a valuable tool for designing sewage outfalls, enabling the selection of an optimal discharge location. The coupling methodology presented involves a dynamical analysis of the sewage plume, with *E. coli* as the reference contaminant, while simultaneously varying metoceanographic parameters that influence this microbiological indicator. A similar approach was used by Morelissen et al. (2013), which provided a good assessment of the environmental impact of the sewage plume in coastal waters. Thus, the *E. coli* plume concentration is evaluated over time because the decay rates of this microorganism are influenced by temperature, salinity, and solar radiation, which vary with the plume’s position within the water column.

The current pattern in the modelled domain rules the fate of the sewage plume. Thus, a good agreement between computed and measured current values is essential for determining the optimal location for sewage discharge. The calculated currents presented a good agreement with field data.

According to Figs. 7 and 8, *E. coli* concentrations correlate strongly with solar radiation levels over the plume (Fig. 6), thereby regulating *E. coli* decay rates. During the daytime, submerged conditions (10 m depth, frame B; 7 m depth, frame C) resulted in longer *E. coli* concentration plume extents. The slight differences observed between these two frames are due to lower sunlight levels at 10 m than at 7 m. However, it is essential to note that during the daytime and under submerged conditions, the plume extensions are significantly longer than in surface plume conditions (frames A and D), which are subjected to higher levels of solar radiation. Submerged plumes resembled the dark conditions shown in Frames E and F, indicating that the depth of the *E. coli* plume can significantly attenuate sunlight along the water column. In the absence of sunlight, salinity and temperature regulate the die-off of *E. coli*. Under such circumstances, the modelled decay rates ranged from 1.34 to 1.63 day^−1^ and were in good agreement with experimental and field studies (Bellair et al. 1977; Mancini 1978) and fairly matched the findings of *E. coli* decay rates in laboratory studies (Wong et al. 2022). Although evaluating *E. coli* plumes in field campaigns has been logistically and economically challenging due to large spatial scales and the influence of dynamic environmental factors, bacterial decay rates have been used indirectly to compare field and model results. The bacterial decay rates modelled in the present work showed a significant agreement with field studies performed in marine waters (Bellair et al. 1977; Canteras et al. 1995; Guillaud et al. 1997; Roberts and Villegas 2017) and align with modern frameworks assessing microbiological risks and persistence in urbanised coastal zones (Erb et al. 2024; Trog-Ferreira et al. 2025).

In both locational alternatives, the *E. coli* plumes extended along the coast, oscillating more frequently from east to west under usual weather conditions and from west to east under bad weather conditions. Based on an analysis performed by Pedrosa de Macena et al. (2023), field samples taken 1 km away on both sides of the effluent discharge point of the Barra da Tijuca submarine outfall indicated unsuitable bathing conditions, corroborating this pattern. Pollution hotspots along the coast are limited to areas around channels that flow into the shore. (Pedrosa de Macena et al. 2023). A similar trend was also observed in a previous study conducted near the Ipanema outfall diffuser (Schaffel et al. 1990).

Figure 9 delimits areas parallel to the coast, where the percentage of unsuitable bathing conditions decreases with the distance from the effluent discharge point. The two locational options differ in that the alternative eliminates the risk of unsuitable bathing conditions in the Maricas Islands. Neither locational alternative (axis A or B) compromises bathing conditions in the beach zone, corroborating field studies that indicate that marine sewage outfalls do not contaminate the beach zone (Mamidisetti & Vijay 2023; Pedrosa de Macena et al. 2023; Schaffel et al. 1990). In these cases, contamination results from the discharge of polluted inland waters into coastal waters or from stormwater runoff (Nadella and Sen 2022; Adolf et al. 2023). Rather than being a problem, studies revealed that coastal water quality improves once sewage outfalls begin operating. In Sydney, Australia, a significant improvement in the water quality of nearby beaches was observed after the opening of the submarine outfalls of Malabar, North Head, and Bondi (Fagan et al. 1992; Besley and Birch 2019; Manning et al. 2019), corroborating that when properly designed sewage outfalls have a significant role in preventing, rather than causing, the pollution of beach zones.

It is important to highlight that, even though the modelling framework is robust, several sources of uncertainty and inherent limitations must be acknowledged to contextualise the findings.

The 31-day simulation period (July 2022) was chosen to represent a critical winter scenario. Even though the hydrodynamic model has been validated with high-frequency ADCP data, the results are subject to the specific synoptic meteorological events of that window. Longer-term simulations or the inclusion of extreme storm surge events could further refine the probabilistic risk intervals, although the current results provide a representative baseline for typical seasonal circulation.

The model uses hourly net radiation data at the free surface as a primary driver of bacterial decay, effectively incorporating variability in cloud cover and atmospheric attenuation over the simulated period. However, uncertainty remains regarding the vertical light extinction coefficient (*K*_*e*_). This parameter, which dictates light extinction with depth, was supposed to be constant based on regional Secchi depth averages (7.6 m). *K*_*e*_ can fluctuate due to transient changes in turbidity, which may locally alter the bacterial decay rates within the submerged plume.

*E. coli* decay is modelled using empirical formulations that consider the combined effects of radiation, salinity, and temperature. Although these formulations have been referenced in coastal engineering, bacterial decay rates can vary with the specific bacterial strain and the presence of predatory microorganisms or organic matter in the effluent, which were not explicitly modelled. However, it is worth highlighting that not considering predation in modelling has two reasons. The first concerns the complexity of gathering input data regarding the type, concentrations, and especially, the spatial distribution of these predatory microorganisms in the modelled domain. The second reason lies in the conservative aspect. Considering predation in the modelling would reduce the *E. coli* concentration, thereby minimising risks, whereas some strains of predatory microorganisms could pose a sanitary risk in bathing waters.

**A** primary limitation is the absence of direct microbiological field validation for the effluent plume. Since ITSO is a projected facility, it is impossible to measure real-time *E. coli* concentrations in the water column under operating conditions. To mitigate this, the study relies on a preliminary approach, validating the hydrodynamic transport (as shown in Fig. 4) and applying established, peer-reviewed biological decay constants. This predictive approach is standard for environmental licensing and provides a high-confidence proxy for pre-operational impact assessment.

## Conclusion

Modelling the environmental assessment of sewage plumes has become a powerful tool for designing optimal pipeline lengths and effluent discharge points, as well as predicting their fate before outfall operation begins. The coupling methodology presented integrates hydrodynamic, near-field, far-field, and bacterial decay models, enabling a more realistic and dynamic representation of outfall *E. coli* plumes by accounting for simultaneous variations in the key environmental parameters. For the two locational alternatives considered, *E. coli* concentrations presented a significant correlation with solar radiation levels within the plume. At night, decay rates vary with salinity and temperature, leading to higher background concentrations before sunrise due to reduced die-off. In the environmental impact assessment of ITSO, the results indicated that both alternatives maintain shoreline water quality within bathing standards, corroborating that well-designed submarine outfalls do not contaminate beach zones.

The main goal of this study lies in the strategic optimisation of the outfall location. While the original design posed sporadic risks to Marica’s Islands, the alternative location eliminated this environmental threat. Furthermore, this relocation allowed for a 1175-m reduction in pipeline length, yielding significant economic savings without compromising environmental safety. This outcome demonstrates the efficiency of the proposed modelling framework as a decision-support tool for maximising both environmental protection and cost-effectiveness in coastal sanitation projects.

## Data Availability

The data that support the findings of this study are available upon request.
